# Preliminary Evaluation of the Circle Sequencing Task (CST) as a Cognitive–Motor Tool for Concussion Assessment

**DOI:** 10.3390/sports14070269

**Published:** 2026-06-29

**Authors:** Tyler Moore, Kirsty Brock, Jac Palmer, Ryan Baker, Naser Taleshi, Genevieve Williams

**Affiliations:** 1 Exeter Head Impact, Brain Injury and Trauma (EXHIBIT) Research Group, Public Health and Sport Sciences, University of Exeter, Exeter EX2 4TH, UK; tm607@exeter.ac.uk (T.M.); k.brock@exeter.ac.uk (K.B.); jp955@exeter.ac.uk (J.P.); rb825@exeter.ac.uk (R.B.);; 2 School of Sport, Physical Education & Coaching, Plymouth Marjon University, Plymouth PL6 8BH, UK; 3 Cardiff School of Sport, Cardiff Metropolitan University, Cardiff CF23 6XD, UK

**Keywords:** brain, concussion, HIA, mild traumatic brain injury (mTBI)

## Abstract

Current pitchside concussion assessments are limited by low sensitivity, reliance on player self-report, and the need for on-site healthcare professionals. Impairments in cognitive function and motor control are a key predictor of concussive injury. The Circle Sequencing Task (CST) is a newly developed concussion assessment tool to assess both cognitive function and motor control in one test. Here, we seek to assess the CST’s ability to differentiate between healthy and recently concussed individuals relative to existing cognitive tests. Concussed (*n* = 13; mean age 26 ± 7 yrs) and healthy (*n* = 13; mean age 28 ± 9 yrs) participants completed the CST, the Trail Making Test, a Go/No-Go task, the Digit Span Test and a simple reaction time task online via a link. Concussed individuals showed significant deficits in inhibitory control (*p* = 0.01) and memory (*p* = 0.04) components of the CST compared to healthy controls, with these components showing a larger effect size (d = 1.05 and d = 0.78, respectively) than metrics derived from the existing cognitive tests of Go/No-Go (d = 0.37) and the Digit Span Test (d = 0.52). The findings provide preliminary evidence that CST-derived inhibitory control and memory metrics may differ between recently concussed individuals and healthy controls and warrant further validation in larger clinically controlled studies.

## 1. Introduction

Detecting a concussion is essential to prevent the immediate and delayed risks associated with large traumatic forces applied to the head. If undetected, there is an increased risk to cognitive, neurological, and mental health. Effective pitchside assessment is critical in detecting sustained or transient shifts from normal cerebral physiology [1]. Notwithstanding the complex neuropathophysiological processes at play in the acute period following a concussion, the logistical and time-pressured nature of sport adds to the challenge of robust and timely concussion assessment [2,3,4].

Several standardised concussion assessments are commonly used post-game or during a head injury assessment window, such as the Sport Concussion Assessment Tool—now in its sixth iteration (SCAT-6) [5]—Immediate Post-Concussion Assessment and Cognitive Testing (ImPACT) [6], and CogSport [7]. Of these, only the SCAT contains an immediate assessment element suitable for pitchside testing, which has been endorsed for use by global sporting bodies. The crux of current concussion assessments places a strong emphasis on player self-report of symptoms as well as tests of basic orientation, such as the Maddocks questions, which assess awareness of recent events. While these tools are essential components of concussion evaluation, they are limited by their susceptibility to player manipulation, language barriers, and the requirement for administration by trained medical professionals [8,9,10,11]. In response to these limitations, there has been a growing shift toward incorporating objective, quantitative cognitive measures to complement existing assessment practices [12,13,14,15].

Cognitive and neuropsychological testing provides critical insight into head-impact-related alterations in brain function [16] and has been shown to predict recovery trajectories following both traumatic brain injury [17,18,19] and mild traumatic brain injury (mTBI) [20,21]. Digitised cognitive assessments, in particular, offer the potential for rapid administration in time-limited settings, such as pitchside environments [22,23], while also reducing reliance on specialist medical personnel. However, the translation of these tools to concussion assessment has been minimal, with concerns remaining regarding their sensitivity, specificity, reliability, and the speed with which meaningful results can be obtained [24].

Sport-related concussion assessment is an iterative and evolving field [25]. Techniques assessing fine motor control represent a promising area of investigation because they rely on neural systems involved in visuomotor integration, coordination, and executive function, including the primary sensory and motor cortices, rostral cingulate motor area, premotor cortex, and the parietal and prefrontal cortices [26,27]. Consistent with the network-level dysregulation hypothesis, concussion may disrupt communication within frontoparietal and sensorimotor networks, resulting in impairments in inhibitory control, visuomotor coordination, motor sequencing, and working memory [28]. These facets underpin the multidomain structure of computer-based assessments, which may provide a sensitive means of detecting subtle cognitive–motor dysfunction. Accordingly, the present study evaluates the preliminary discriminative ability of the CST following sport-related concussion.

There have been multiple notable developments in computer-based assessment in recent years [21,29,30,31,32,33,34,35]. This work describes a cognitive–motor integration (CMI) task in which participants perform centre–out movements to four static targets, allowing fine motor control to be empirically quantified. Manipulating cognitive constituents such as memory and decoupling the cursor movement for nonstandard visuomotor mapping are used to increase task difficulty. Studies have found deficits in adults [21,25,36] and adolescents [30,31,32] with a history of concussion across different CMI conditions, with greater differences seen in the nonstandard visuomotor mapping task—where cursor movement is decoupled. Elite hockey players who had experienced a concussion during a season exhibited deficits in reaction times, movement times, and accuracy [33]. Despite these promising results, findings have only been replicated in lab conditions with specialised equipment and after a vast period of time since the concussion occurred in participants. It remains unclear how testing in more ecologically valid environments, such as at pitchside or on a person’s phone or laptop, outside the control of laboratory conditions, could influence the ability to distinguish concussed from non-concussed individuals in these types of tasks.

This work attempts to extend the foundations of the CMI task by introducing further cognitive elements from several popular cognitive tests, namely, Go/No-Go, the Trail Making Task, Simple Reaction Time, and Digit Span. In addition, this work looks to integrate an app-based approach to concussion assessment that does not require specialist equipment such as multiple touch screens or capacitive-touch gloves, which are not suitable for remote or pitchside testing. The Circle Sequencing Task (CST) is a brief, language-neutral CMI test designed to address the limitations of existing tests by utilising a design that can be implemented on any computer or mobile device, making it flexible and appropriate for rapid pitchside assessment. The CST seeks to effectively detect both subtle and clear concussion symptoms by assessing cognitive and motor functions such as reaction time, motor control, inhibition, memory, and visuomotor skills. Unlike existing tools, the CST can be completed in under 5 min without the need for clinician administration or specialist equipment. This efficient, quantitative tool offers valuable insights into the specific challenges athletes face when returning to play. Although standardised concussion assessment tools such as the SCAT are widely used in clinical and sports settings, no single test is currently regarded as a definitive gold-standard diagnostic tool for concussion [5,37]. Validating a new test solely based on its agreement with the gold standard is not feasible. Instead, researchers must rely on convergent validity with related tests to ascertain whether the new assessment aligns with the existing methods [38]. As the Circle Sequencing Task (CST) is a newly developed assessment tool, this study aims to confirm its efficacy in distinguishing concussed versus non-concussed individuals relative to existing cognitive tests.

## 2. Materials and Methods

### 2.1. Participants

The sample for this study consisted of 13 concussed (7 male, 3 female, 3 non-binary; mean age 26 ± 7 years) and 13 healthy controls (HCs; 8 male, 5 female; mean age 28 ± 9 yrs). Individuals with a concussion were self-diagnosed (*n* = 4) or diagnosed by a clinical professional (*n* = 9) within 10 days (mean time = 8 ± 2 days) of testing. These values are shown in Table 1. Given that participant recruitment occurred in a collegiate sporting population and outside of a clinical setting, sample availability was constrained. Self-diagnosed participants completed a questionnaire adapted from the Rivermead Post-Concussion Symptoms Questionnaire [39] and the Philadelphia Head Injury Questionnaire [40] to ensure that reported symptoms aligned with those observed in concussive injuries. Participants were also asked to provide any details about any previous concussions they may have experienced. Participants were asked to self-report any neurodivergence or mental health diagnoses, with a positive self-report leading to their omission from the sample.

### 2.2. Procedure

Participants completed testing remotely using their own devices and accessed the tests via an email link. To participate, a computer or laptop with a mouse was required to record responses. Participants were given instructions to find a quiet location with minimal distractions and close any applications that may interfere with the testing software. It should be acknowledged that the procedural focus on ecological validity and human interface with technology beyond the laboratory environment created several methodological limitations. Participants completed the testing protocol with an external computer mouse on their personal desktop or laptop devices. The use of heterogeneous personal devices does introduce potential sources of error in the form of measurement precision and latency that are discussed in detail later in this paper.

### 2.3. Measures

**Figure 1:**
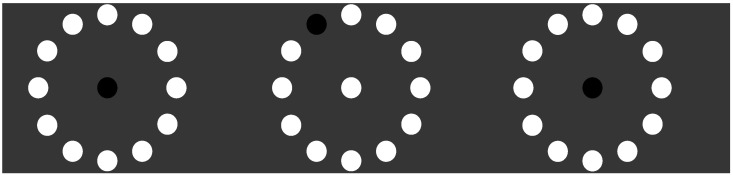
The basic layout of the Circle Sequencing Task, where 12 targets are presented, and across the trials the participant must move to one highlighted target before moving back to the centre.

The cognitive battery was developed by adapting previously validated assessments from the open-access Pavlovia directory, with existing paradigms edited minimally in terms of timings and user interface [41]. The CST was split into 3 distinct blocks, namely, guided, inhibition, and memory trials, with a familiarisation block at the beginning. Each block involved a randomly generated four-element sequence that the participant must interact with. The participant was required to move their mouse between targets that turned from white to black in a sequence via the most direct and accurate route until completion. Firstly, there was a familiarisation trial designed to help participants become familiar with the task and the main movement patterns utilised, with no data recorded. Participants were asked to move from the centre to any highlighted black targets and avoid any red targets, which prepared them for the first two main blocks of the task. The first block, the guided block, required participants to follow a sequence of highlighted targets with their cursor. Targets were defined as a circle turning from white to black. The sequence involved moving from the central target to the outer targets as they turned black and back to the central target again. Each sequence consisted of the presentation of four random targets, where five sequences were performed, resulting in 20 trials for this block. In the second block, the inhibition block, the Go/No-Go element was introduced. Participants were required to consider the colour of the targets that appeared, with a 3:1 ratio of blue to red targets. They were instructed not to move if the red targets appeared and to move as quickly as possible to the blue targets. The red targets remained for 2.5 s before a new target was presented. Similar to the guided block, the inhibition block also contained 20 trials. Lastly, the memory block consisted of two separate elements, the sequence watch and the sequence recall. In the sequence watch, participants watched a four-item sequence being produced, with each target appearing for 1.8 s before moving back to the centre and on to the next target. After all four targets were shown, the sequence recall began, where participants had to accurately recall and reproduce the sequence they had just seen on a blank canvas of targets, shown in Figure 1. An example of CST performance and results output is available at https://doi.org/10.6084/m9.figshare.32687832. Unlike the sequencing and inhibition blocks, only four repeats of the memory trials were used, as it takes more time to watch the sequences being produced, with four repeats taking 28.8 s. Within the CST, RT, movement time, fine motor control, inhibition, memory, and visual search were quantified.

For the Trail Making Task (TMT), participants completed two trials of TMT-A, which contained only numbers, followed by two trials of TMT-B, which contained both numbers and letters. TMT-A trials consisted of 5 and 8 items, respectively, while TMT-B trials were longer with 8 and 16 items. The sequences used the same numbers and letters, from 1 to 8 and A to H, but their locations were randomised in each trial. Errors were not counted, and participants were able to move to the correct answer if they landed on an incorrect one. The primary outcome measures were the time taken to reach each target and complete each trial.

For Go/No-Go, participants were presented with 40 trials where a coloured circle appeared in the centre of the screen. The circles appeared either in blue or orange colour, with a 50% chance of each in a given trial. Participants were instructed to respond as fast as possible by clicking or pressing the space bar when the circle was blue (Go) and to withhold their response when the circle was orange (No-Go). The circle was displayed for 1 s, and a fixation cross appeared for 1 s between each trial. The primary output measures of this task were reaction time and accuracy of response.

Participants were required to watch a series of numbers displayed on the screen, with each digit appearing one at a time for the Digit Span Test (DS). During the task, a fixation cross was shown for 1 s before each digit appeared for another second. After the presentation of the number sequence, a textbox would appear for participants to type in the number sequence that they saw. The task began with a 3-digit number and increased in length by one digit for two consecutive correct answers. However, if the participant made two incorrect guesses, the task would end. The sequences used were consistent across all participants to allow for comparisons. The main output from this task was the Digit Span, which represents the maximum length of the number sequence that the participant could correctly recall. The Digit Span was used to assess the participant’s working memory capacity.

For simple reaction time (SRT), participants were instructed to focus on a white box displayed in the centre of the screen, waiting for a cross to appear. Once the cross appeared, participants were instructed to react as quickly as possible by pressing the space bar. The interstimulus intervals, which referred to the time between the appearance of the cross, were randomly adjusted from a pool of 10 timings, ranging between 1 and 3 s. The purpose of this task was to measure the participant’s simple reaction time, which was the main output of the task.

### 2.4. Data Processing

From the CST, the participants’ reaction time (RT), time to complete (TTC), pathlength (PL), peak velocity (PV), time to stop (TTS), and memory score (MS) were recorded. The TMT provided data on TTC and RT, while the Go/No-Go task measured accuracy and RT. DS length and Simple RT were also documented from the respective tasks. Details of each measurement and their relevant CST block can be found in Table 2. 

### 2.5. Data Analysis

The analysis of the data was carried out using IBM SPSS Statistics version 28.0 (SPSS, Inc., Chicago, IL, USA). Boxplots were used to identify outliers outside of 3 standard deviations, which were subsequently removed from the data set. The majority of variables violated the assumption of normality, as determined by the Kolmogorov–Smirnov test of normality. Non-parametric tests were used, namely, the Mann–Whitney U test. Where the assumption of normality was met, independent samples *t*-tests were used. The alpha value was set at *p* < 0.05. Cohen’s d (95% CI) was used to determine effect size, with d ≤ 0.2 indicating a small effect, d = 0.5 a medium effect, and d ≥ 0.8 a large effect. Data were presented as mean ± standard deviation (sd).

## 3. Results

For the CST, the TTS was significantly slower (U = 38, *p* = 0.01) in concussed individuals (0.08 s ± 0.05 s) than in HCs (0.04 s ± 0.02 s), and MS was significantly lower (U = 48.5, *p* = 0.04) in concussed individuals (2.83 ± 0.82) than in HCs (3.45 ± 0.65) as seen in Table 3. An independent *t*-test revealed no difference for PV (t(24) = −1.15, *p* = 0.26) between concussed individuals (3.04 px/s ± 0.95 px/s) and HCs (3.45 px/s ± 0.86 px/s). PL exhibited comparable values for concussed individuals (0.40 px ± 0.20 px) and HCs (0.40 px ± 0.02 px) with no significant differences (U = 79.5, *p* = 0.79). This was also found across RT (concussed: 0.46 s ± 0.15 s, HC: 0.46 s ± 0.18 s; (U = 77, *p* = 0.70)) and TTC (concussed: 0.74 s ± 0.20 s, HC: 0.64 s ± 0.13 s; (U = 53, *p* = 0.11)). When comparing the cognitive battery, only TTC for TMT-A showed a significant difference (t(24) = 2.37, *p* = 0.03) between concussed (6.71 ± 4.85 s) and HCs (4.85 ± 0.99 s), with concussed individuals taking longer to complete.

Effect sizes were used to compare CST metrics with standard cognitive tests. For the metrics where a significant difference was present between the concussed and non-concussed groups, MS (d = 0.78 (0.74–0.82)) displayed considerably larger effect sizes compared to GNG accuracy (d = 0.37 (0.35–0.39)) and DS length (d = 0.52 (0.49–0.55)). TTS (d = 1.05 (1.00–1.10)) showed the largest effect size overall, whilst TTC (d = 0.59 (0.56–0.62)) displayed a lower, albeit still moderate, effect size compared to TMT-A_TTC (d = 0.94 (0.89–0.99)).

## 4. Discussion

As the Circle Sequencing Task (CST) is a newly developed assessment tool, this study aimed to evaluate whether CST results differ between concussed and non-concussed individuals. When evaluating the CST’s sensitivity to concussion, the results showed that TTS was significantly longer and MS was significantly lower in concussed individuals than in the healthy control group, each with large effect sizes (d = 1.05 and d = 0.78, respectively), indicating that these metrics are sensitive to the effects of concussive injury. These metrics also demonstrated the largest effect sizes of all variables analysed from the CST and existing cognitive tests. For the existing cognitive tests, only TTC for TMT-A was significantly different between the groups, with a large effect size (d = 0.94), whereas Go/No-Go, TMT-B, Simple RT and Digit Span were not significantly different. Interestingly, the TTC for TMT-A showed a larger effect size than its CST counterpart (d = 0.59).

The differences in CST metrics between the concussed and healthy control groups are consistent with existing research. These findings lend support to the proposition [42] that speed and memory factors are the most predictive elements of a cognitive test for concussion. TTS was found to be significantly higher in the concussion group, which is consistent with findings from [43], using a Go/No-Go task, and [44], using a Stroop test and choice response task, suggesting deficits in inhibitory control following a concussion.

It is a common finding that simple RT is affected by a concussion [29,33,45,46,47,48]; however, this was not found to be the case in the current study, with no statistically significant change in RT within the CST. It is possible that the remote nature of testing caused variability in RT scores, as this is the metric most likely to be influenced by differing computer configurations and aspects of concentration and distraction, which is particularly relevant when considering the most appropriate tools for pitchside testing of concussion, where potential for external distraction is high.

MS from the CST was lower in the concussed group compared to the healthy controls. The deficits found in memory scores are in line with previous research on Digit Span length [49], though no statistically significant difference in Digit Span was found in this study. This indicates that the CST memory task may be more robust in detecting concussive injury than existing tasks. This serves as a promising finding that can be used in the development of pitchside and remote concussion assessment.

The most notable deviation from the existing literature was the lack of significance in PL, which was considered a key variable for detecting concussion in prior CMI research [29,50]. This discrepancy could be attributed to three factors: time since injury, task difficulty, and the remote nature of the testing. Specifically, in the research presented by [29], the time since concussion was 18.6 months (SD = 32 months), indicating that participants may have been at different stages of recovery. Similarly, studies by Dalecki et al. [31] and Dalecki et al. [32] included participants who were 12.9 months (SD = 10.4 months) and 14.3 months (SD = 11.3 months), respectively, post-concussive event. This must be considered alongside the standard recovery times for concussion, which can vary but are thought to be between two weeks to one month depending on age and severity of the injury [51], though it should be acknowledged that these timeframes are contested [52]. In previous studies, participants may not have been considered “concussed” at the point of testing, unless they were experiencing persisting post-concussion symptoms. Persisting post-concussion symptoms are characterised by the presence of “any symptom that cannot be attributed to a preexisting condition and that appeared within hours of an mTBI, that is still present every day 3 months after the trauma, and that has an impact on at least one sphere of a person’s life” [53]. In contrast, the current study focused on the acute period of ≤10 days after a concussion, resulting in greater homogeneity in the concussive state. The homogeneity and the temporal proximity of testing to the injury event are significant strengths of the current paper, providing greater generalisability of the findings to a specific injury recovery window as well as the real-world application of pitchside assessment.

Further contrasts with previous research were recorded for TTC in the CST, where times were not found to be significantly different between the concussed and non-concussed groups. Previous studies have found TTC to be a strong predictor of concussion [29,31,32,33,34,35,36,50]. It is unclear whether the low participant numbers contributed to the inconsistencies between this study and previous research. In contrast, our findings for PV were in agreement with previous research. There is an evolving body of literature that demonstrates that PV may not be a suitable measure for detecting concussion in computer-based concussion assessments [31,32,35].

It is essential to acknowledge the limitations of this study, particularly the relatively small sample size of only 13 concussed individuals and corresponding healthy controls. Although the current study did not categorically demonstrate the CST’s sensitivity to concussion, future studies should look to employ the CST with larger samples. The mix of officially diagnosed and self-reported concussions may have detrimentally influenced the findings of this study. Self-reported concussion history may be subject to recall bias or misclassification, potentially resulting in the inclusion of participants who did not meet clinical diagnostic criteria for concussion. Moreover, the remote testing approach, while convenient and feasible for pitchside use in sports, could have introduced variability due to the lack of control over the participants’ testing environments. It is also important to note that the remote data collection did not capture information about the participants’ hardware or software, including factors such as screen resolution, internet browser, or mouse speed. Testing conducted on heterogeneous personal devices is subject to multiple sources of variability, most notably mouse polling rate (125–1000 Hz) and display refresh rate (60 Hz vs. 120 Hz). These variables have been shown to potentially influence how individuals interact with the stimuli and the timing of a computer-based task [53]. While Bridges et al. [54] argue that PsychoPy is one of the most precise and lowest-latency online testing platforms, it is important to acknowledge that there might still be some influence from these factors that would not be present in a controlled laboratory setting. Based on the preliminary nature of this work, we highlight that we cannot infer diagnostic utility or clinical applicability, and there is limited external validity of the metrics.

There are several directions for future research incorporating the CST and facilitating its further development. Primarily, there is a need for a large-scale study with a more diverse participant pool. Such an extensive investigation would serve to validate the efficacy of the CST tool more comprehensively while simultaneously bolstering confidence in its diagnostic utility and ability to track functional change across the recovery period. Work should also be conducted to assess the validity of the CST over time to ensure it is suitable for real-world application.

## 5. Conclusions

These findings suggest that the CST shows promise as a quantitative concussion assessment tool and highlight the importance of speed and memory factors in cognitive tests for concussion detection. CST metrics did show differences in the presence of concussion, particularly TTS, which was significantly longer and MS, which was significantly lower in the concussed group than in the healthy control group. Overall, these preliminary findings indicate that the CST could be implemented to detect cognitive deficits associated with concussion both remotely and in acute sport settings.

## Figures and Tables

**Table 1:** Participant characteristics. Relevant data is presented as mean ± standard deviation.

Group	*n*	Age (Years)	Time Since Concussion (Days)	Self-Diagnosed Concussion	Gender
Concussed	13	26 ± 7	8 ± 2	4	7 M 3 F 3 NB
Control	13	28 ± 9	n/a	n/a	8 M 5 F

**Table 2:** Each circle sequencing (CST) and cognitive battery metric used throughout analysis.

Measurement	Description	Relevant CST Block
Reaction time (RT; s)	The time between stimulus presentation and initial movement towards target.	Sequencing
Time to Complete (TTC; s)	The time between the sequence starting and the participant entering the final target of each four-element sequence.	Sequencing
Time to Stop (TTS; s)	The first time point that the participant is not moving following presentation of a No-Go (red) target. With a possible value of 0 if no movement occurs.	Inhibition
Peak Velocity (PV; pixel/s)	Maximum velocity obtained during the movement between targets.	Sequencing
Pathlength (PL; pixels)	The distance travelled between the first movement and the movement’s endpoint, calculated via Pythagorean theorem.	Sequencing and Memory
Memory Score (MS; total out of 4)	The number of targets correctly recalled.	Memory
Trail Making Task—A, Time to Complete (TMT-A_TTC; s)	The time taken to complete the TMT-A section.	Cognitive Battery
Trail Making Task—B, Time to Complete (TMT-B_TTC; s)	The time taken to complete the TMT-B section.	Cognitive Battery
Go/No-Go Accuracy (GNG accuracy)	The number of correct responses (assigned 1; incorrect assigned 0) divided by the total number of Go/No-Go trials.	Cognitive Battery
Go/No-Go Reaction Time (GNG_RT; s)	The time taken to start to move to a Go stimulus.	Cognitive Battery
Digit Span length (DS length)	The length of the last correctly recalled number sequence.	Cognitive Battery
Simple Reaction time (Simple RT; s)	The time between the presentation of the stimulus and a button click.	Cognitive Battery

**Table 3:** Mean (±sd) values for the Circle Sequencing Task performance metrics (peak velocity (PV), pathlength (PL), reaction time (RT), time to complete (TTC), time to stop (TTS), memory score (MS)) in the concussed and healthy control groups.

Metric	Concussed	Healthy Controls
PV (px/s)	3.04 ± 0.95	3.45 ± 0.86
PL (px)	0.40 ± 0.20	0.40 ± 0.02
RT (s)	0.46 ± 0.15	0.46 ± 0.18
TTC (s)	0.74 ± 0.20	0.64 ± 0.13
TTS (s)	0.08 ± 0.05	0.04 ± 0.02
MS (out of 4)	2.83 ± 0.92	3.45 ± 0.65

## Data Availability

The datasets generated during and/or analysed during the current study are available from the corresponding author on reasonable request.

## Abbreviations

The following abbreviations are used in this manuscript:

CMI	Cognitive Motor Integration
CST	Circle Sequencing Task
DS	Digit Span
GNG	Go/No Go
HCs	Healthy Controls
ImPACT	Immediate Post-Concussion Assessment and Cognitive Testing
mTBI	mild traumatic brain injury
MS	Memory Score
PL	Path Length
PV	Peak Velocity
REST	Research Engagement Survey Tool
RT	Reaction Time
SCAT	Sport Concussion Assessment Tool
SPSS	Statistical Package for the Social Sciences
SRT	Simple Reaction Time
TBI	Traumatic Brain Injury
TMT	Trail Making Task
TTC	Time to Complete
TTS	Time to Stop

## References

[1] Yue J.K., Phelps R.R.L., Chandra A., Winkler E.A., Manley G.T., Berger M.S. (2020). Sideline concussion assessment: The current state of the art. Neurosurgery.

[2] Broglio S.P., Vagnozzi R., Sabin M., Signoretti S., Tavazzi B., Lazzarino G. (2010). Concussion occurrence and knowledge in Italian football (soccer). J. Sports Sci. Med..

[3] Fraas M.R., Coughlan G.F., Hart E.C., McCarthy C. (2014). Concussion history and reporting rates in elite Irish rugby union players. Phys. Ther. Sport.

[4] Delaney J.S., Caron J.G., Correa J.A., Bloom G.A. (2018). Why Professional Football Players Chose Not to Reveal Their Concussion Symptoms During a Practice or Game. Clin. J. Sport Med..

[5] Echemendia R.J., Brett B.L., Broglio S., Davis G.A., Giza C.C., Guskiewicz K.M., Harmon K.G., Herring S., Howell D.R., Master C.L. (2023). Introducing the Sport Concussion Assessment Tool 6 (SCAT6). Br. J. Sports Med..

[6] Elbin R.J., Fazio-Sumrok V., Anderson M.N., D’Amico N.R., Said A., Grossel A., Schatz P., Lipinski D., Womble M. (2019). Evaluating the suitability of the Immediate Post-Concussion Assessment and Cognitive Testing (ImPACT) computerized neurocognitive battery for short-term, serial assessment of neurocognitive functioning. J. Clin. Neurosci..

[7] Westerman R., Darby D., Maruff P., Collie A. (2001). Computer-assisted cognitive function assessment of pilots. Aust. Def. Force Health.

[8] Alsalaheen B., Stockdale K., Pechumer D., Broglio S.P. (2016). Measurement Error in the Immediate Postconcussion Assessment and Cognitive Testing (ImPACT): Systematic Review. J. Head Trauma Rehabil..

[9] Alsalaheen B., Stockdale K., Pechumer D., Broglio S.P. (2016). Validity of the Immediate Post Concussion Assessment and Cognitive Testing (ImPACT). Sports Med..

[10] Doan B.K., Heaton K.J., Self B.P., Butler Samuels M.A., Adam G.E. (2022). Quantifying head impacts and neurocognitive performance in collegiate boxers. J. Sports Sci..

[11] Powell D., Stuart S., Godfrey A. (2021). Sports related concussion: An emerging era in digital sports technology. npj Digit. Med..

[12] Daly E., Pearce A.J., Finnegan E., Cooney C., McDonagh M., Scully G., McCann M., Doherty R., White A., Phelan S. (2022). An assessment of current concussion identification and diagnosis methods in sports settings: A systematic review. BMC Sports Sci. Med. Rehabil..

[13] Farnsworth J.L., Dargo L., Ragan B.G., Kang M. (2017). Reliability of Computerized Neurocognitive Tests for Concussion Assessment: A Meta-Analysis. J. Athl. Train..

[14] McCrea M., Iverson G.L., Echemendia R.J., Makdissi M., Raftery M. (2013). Day of injury assessment of sport-related concussion. Br. J. Sports Med..

[15] Palmer J.L., Baker R., Regardsoe A., Irwin G., Williams G. (2026). Perceptions of concussion management and testing among healthcare professionals in rugby union: A qualitative analysis. BMJ Open Sport Exerc. Med..

[16] Faria C.d.A., Alves H.V.D., Charchat-Fichman H. (2015). The most frequently used tests for assessing executive functions in aging. Dement. Neuropsychol..

[17] Harvey P.D. (2012). Clinical applications of neuropsychological assessment. Dialogues Clin. Neurosci..

[18] Ord J.S., Greve K.W., Bianchini K.J., Aguerrevere L.E. (2010). Executive dysfunction in traumatic brain injury: The effects of injury severity and effort on the Wisconsin Card Sorting Test. J. Clin. Exp. Neuropsychol..

[19] Woods D.L., Wyma J.M., Herron T.J., Yund E.W. (2016). Computerized Analysis of Verbal Fluency: Normative Data and the Effects of Repeated Testing, Simulated Malingering, and Traumatic Brain Injury. PLoS ONE.

[20] Arciniega H., Shires J., Furlong S., Kilgore-Gomez A., Cerreta A., Murray N.G., Berryhill M.E. (2021). Impaired visual working memory and reduced connectivity in undergraduates with a history of mild traumatic brain injury. Sci. Rep..

[21] Sergio L.E., Gorbet D.J., Adams M.S., Dobney D.M. (2020). The Effects of mild traumatic brain injury on Cognitive-Motor Integration for Skilled Performance [Perspective]. Front. Neurol..

[22] Galetta K.M., Brandes L.E., Maki K., Dziemianowicz M.S., Laudano E., Allen M., Lawler K., Sennett B., Wiebe D., Devick S. (2011). The King-Devick test and sports-related concussion: Study of a rapid visual screening tool in a collegiate cohort. J. Neurol. Sci..

[23] Richardson C., Atkins S., Hurst H., Quinn M., Sinclair J. (2017). Executive function testing to assist identification of pitch-side concussion in elite rugby players. Lancet.

[24] Scharfen H.E., Memmert D. (2019). Measurement of cognitive functions in experts and elite athletes: A meta-analytic review. Appl. Cogn. Psychol..

[25] Falvey É., Tucker R., Fuller G., Raftery M. (2021). Head injury assessment in rugby union: Clinical judgement guidelines. BMJ Open Sport Exerc. Med..

[26] Ehrsson H.H., Fagergren A., Jonsson T., Westling G., Johansson R.S., Forssberg H. (2000). Cortical activity in precision-versus power-grip tasks: An fMRI study. J. Neurophysiol..

[27] Prodoehl J., Corcos D.M., Vaillancourt D.E. (2009). Basal ganglia mechanisms underlying precision grip force control. Neurosci. Biobehav. Rev..

[28] Kenzie E.S., Parks E.L., Bigler E.D., Wright D.W., Lim M.M., Chesnutt J.C., Hawryluk G.W., Gordon W., Wakeland W. (2018). The dynamics of concussion: Mapping pathophysiology, persistence, and recovery with causal-loop diagramming. Front. Neurol..

[29] Brown J.A., Dalecki M., Hughes C., Macpherson A.K., Sergio L.E. (2015). Cognitive-motor integration deficits in young adult athletes following concussion. BMC Sports Sci. Med. Rehabil..

[30] Caffey A.L., Dalecki M. (2021). Evidence of residual cognitive deficits in young adults with a concussion history from adolescence. Brain Res..

[31] Dalecki M., Albines D., Macpherson A., Sergio L.E. (2016). Prolonged cognitive–motor impairments in children and adolescents with a history of concussion. Concussion.

[32] Dalecki M., Gorbet D.J., Macpherson A., Sergio L.E. (2019). Sport experience is correlated with complex motor skill recovery in youth following concussion. Eur. J. Sport Sci..

[33] Hurtubise J., Gorbet D., Hamandi Y., Macpherson A., Sergio L. (2016). The effect of concussion history on cognitive-motor integration in elite hockey players. Concussion.

[34] Hurtubise J., Gorbet D., Hughes C., Macpherson A., Sergio L. (2017). White matter integrity and its relationship to cognitive-motor integration in females with post-concussion syndrome. Br. J. Sports Med..

[35] Smeha N., Kalkat R., Sergio L.E., Hynes L.M. (2022). Sex-related differences in visuomotor skill recovery following concussion in working-aged adults. BMC Sports Sci. Med. Rehabil..

[36] Echlin H.V., Gorbet D.J., Sergio L.E. (2020). Assessment of a Cognitive-Motor Training Program in Adults at Risk for Developing Dementia. Can. Geriatr. J..

[37] Ellis M.J., Russell K. (2019). The Potential of Telemedicine to Improve Pediatric Concussion Care in Rural and Remote Communities in Canada [Hypothesis and Theory]. Front. Neurol..

[38] Goodman M.S., Ackermann N., Haskell-Craig Z., Jackson S., Bowen D.J., Sanders Thompson V.L. (2022). Construct validation of the Research Engagement Survey Tool (REST). Res. Involv. Engagem..

[39] King N.S., Crawford S., Wenden F.J., Moss N.E., Wade D.T. (1995). The Rivermead Post Concussion Symptoms Questionnaire: A measure of symptoms commonly experienced after head injury and its reliability. J. Neurol..

[40] Curry L.M., Ivins R.G., Gowen T.L. (1991). Philadelphia Head Injury Questionnaire.

[41] Peirce J., Gray J.R., Simpson S., MacAskill M., Höchenberger R., Sogo H., Kastman E., Lindeløv J.K. (2019). PsychoPy2: Experiments in behavior made easy. Behav. Res. Methods.

[42] Schatz P., Maerlender A. (2013). A Two-Factor Theory for Concussion Assessment Using ImPACT: Memory and Speed. Arch. Clin. Neuropsychol..

[43] Kontos A.P., Reches A., Elbin R.J., Dickman D., Laufer I., Geva A.B., Shacham G., Dewolf R., Collins M.W. (2016). Preliminary evidence of reduced brain network activation in patients with post-traumatic migraine following concussion. Brain Imaging Behav..

[44] Xu B., Sandrini M., Levy S., Volochayev R., Awosika O., Butman J.A., Pham D.L., Cohen L.G. (2017). Lasting deficit in inhibitory control with mild traumatic brain injury. Sci. Rep..

[45] Coenen J., Henckert S., Lausberg H., Helmich I. (2022). Post-concussion symptoms and clinical reaction time performance of athletes with a history of concussion. J. Sports Med. Phys. Fit..

[46] Del Rossi G. (2017). Evaluating the Recovery Curve for Clinically Assessed Reaction Time After Concussion. J. Athl. Train..

[47] Lempke L.B., Howell D.R., Eckner J.T., Lynall R.C. (2020). Examination of Reaction Time Deficits Following Concussion: A Systematic Review and Meta-analysis. Sports Med..

[48] Locklin J., Bunn L., Roy E., Danckert J. (2010). Measuring Deficits in Visually Guided Action Post-Concussion. Sports Med..

[49] Ashton J., Coyles G., Malone J.J., Roberts J.W. (2021). Immediate effects of an acute bout of repeated soccer heading on cognitive performance. Sci. Med. Footb..

[50] Kelty-Stephen D.G., Qureshi Ahmad M., Stirling L. (2015). Use of a tracing task to assess visuomotor performance for evidence of concussion and recuperation. Psychol. Assess..

[51] Williams R.M., Puetz T.W., Giza C.C., Broglio S.P. (2015). Concussion Recovery Time Among High School and Collegiate Athletes: A Systematic Review and Meta-Analysis. Sports Med..

[52] Lagacé-Legendre C., Boucher V., Robert S., Tardif P.-A., Ouellet M.-C., de Guise E., Boulard G., Frémont P., Émond M., Moore L. (2021). Persistent Postconcussion Symptoms: An Expert Consensus-Based Definition Using the Delphi Method. J. Head Trauma Rehabil..

[53] Kamins J., Bigler E., Covassin T., Henry L., Kemp S., Leddy J.J., Mayer A., McCrea M., Prins M., Schneider K.J. (2017). What is the physiological time to recovery after concussion? A systematic review. Br. J. Sports Med..

[54] Bridges D., Pitiot A., MacAskill M.R., Peirce J.W. (2020). The timing mega-study: Comparing a range of experiment generators, both lab-based and online. PeerJ.

